# Surgical removal of Waldeyer’s ring and long-term risk of Sjögren’s syndrome: a population-based cohort study investigating the mucosal immune link

**DOI:** 10.3389/fimmu.2026.1760386

**Published:** 2026-02-05

**Authors:** Hua-Wei Chi, Chia-Hung Su, Yu-Hsun Wang, Chun-Hou Liao, Mingchih Chen, Jeng-Wen Chen, James Cheng-Chung Wei

**Affiliations:** 1 Department of Otolaryngology–Head and Neck Surgery, Cardinal Tien Hospital and Fu Jen Catholic University, New Taipei, Taiwan; 2 Department of Otolaryngology–Head and Neck Surgery, National Taiwan University Hospital, Taipei, Taiwan; 3 Department of Medical Research, Chung Shan Medical University Hospital, Taichung, Taiwan; 4 Division of Urology, Department of Surgery, Cardinal Tien Hospital and School of Medicine, Fu-Jen Catholic University, New Taipei, Taiwan; 5 Department of Hospital Management, Graduate Institute of Business Administration, Fu Jen Catholic University, New Taipei, Taiwan; 6 Artificial Intelligence Development Center, Fu Jen Catholic University, New Taipei, Taiwan; 7 Department of Allergy, Immunology & Rheumatology, Chung Shan Medical University Hospital, Taichung, Taiwan; 8 Graduate Institute of Integrated Medicine, China Medical University, Taichung, Taiwan; 9 Institute of Medicine/Department of Nursing, Chung Shan Medical University, Taichung, Taiwan

**Keywords:** adenoidectomy, autoimmune disease, immune tolerance, Sjögren’s syndrome, tonsillectomy, TriNetX

## Abstract

**Background:**

The palatine tonsils and adenoids are critical inductive sites for the mucosa-associated lymphoid tissue (MALT) system, educating B cells for salivary defense. While their removal is common, the long-term impact on mucosal autoimmunity remains unclear. We hypothesized that disrupting this lymphoid network increases susceptibility to Sjögren’s syndrome (SS).

**Methods:**

Using the TriNetX US Collaborative Network (2006–2023), we conducted a retrospective cohort study matching 302,737 patients who underwent tonsillectomy/adenoidectomy with 302,737 non-surgical controls (propensity score-matched 1:1). Hazard ratios (HR) for incident SS were estimated using Cox proportional hazards models.

**Results:**

The surgical cohort exhibited a significantly elevated risk of SS (HR 1.52; 95% CI 1.23–1.87; p<0.001) compared to controls. This risk was most profound in individuals operated on before age 18 (HR 2.27) and in African American patients (HR 2.26). Sensitivity analyses using negative outcome controls (e.g., burns, injuries) confirmed the specificity of the association.

**Conclusion:**

Tonsillectomy is independently associated with an increased risk of SS. These findings suggest that early-life removal of nasopharyngeal lymphoid tissue may disrupt the MALT axis, impairing oral tolerance and predisposing to salivary autoimmunity.

## 1 Introduction

Tonsillectomy and adenoidectomy are among the most frequently performed surgical procedures in children and young adults (1, 2), primarily indicated for recurrent tonsillitis, peritonsillar abscesses, sleep-disordered breathing, and obstructive airway disease (3, 4). Although generally considered safe and effective for symptom relief (5–7), the removal of tonsillar and adenoidal tissues has important implications for mucosal and systemic immune function (8). The palatine tonsils and adenoids constitute major components of Waldeyer’s ring and serve as inductive sites for antigen sampling, B-cell maturation, and mucosal immunoglobulin production. These lymphoid structures play a central role in shaping early-life immune tolerance, regulating T–B cell interactions, and maintaining homeostasis within the upper aerodigestive mucosal barrier (9, 10). Disruption of these tissues during critical developmental periods may therefore alter long-term immune equilibrium, yet the magnitude and clinical significance of this effect remain incompletely understood.

Recent epidemiologic studies have raised concerns that the removal of lymphoid organs during childhood may predispose individuals to subsequent immune dysregulation. Large population analyses have suggested associations between tonsillectomy and increased risks of allergic diseases, respiratory infections, and several autoimmune disorders (11). These findings support a broader hypothesis that lymphoid tissue excision may perturb mucosal-associated lymphoid tissue (MALT) function and diminish the regulatory pathways essential for maintaining tolerance to self-antigens. However, existing evidence is inconsistent (12), often limited by small sample sizes, inadequate control groups, and insufficient adjustment for confounding variables such as infection burden, demographic differences, and preexisting comorbidities.

Sjögren’s syndrome (SS) is a chronic, systemic autoimmune disease characterized by lymphocytic infiltration of exocrine glands, leading to xerostomia, keratoconjunctivitis sicca, and diverse extraglandular manifestations (13, 14). The disease predominantly affects middle-aged women, with an estimated female-to-male ratio of 9:1 (15, 16). While the precise pathogenesis of SS remains elusive, aberrant activation of mucosal immunity, impaired tolerance mechanisms, and dysregulated epithelial–lymphoid interactions are thought to play important roles (17, 18). Because tonsils and adenoids are critical sites for early immune education and IgA-mediated mucosal defense, their removal may influence downstream pathways implicated in the development of SS (13, 19, 20). Nonetheless, evidence directly linking tonsillectomy or adenoidectomy to later SS risk has been scarce. No prior study has systematically evaluated this association using modern real-world data networks, nor examined whether age, sex, ethnicity, or metabolic factors modify this potential relationship.

Physiologically, the tonsils and salivary glands share a functional “mucosal bridge.” Tonsils act as the primary inductive site where naive B cells are exposed to oral antigens and differentiate into IgA-producing plasmablasts (21–23). These cells express specific homing receptors (e.g., C-C chemokine receptor 9, C-C chemokine receptor 10) allowing them to migrate to effector sites, including the salivary and lacrimal glands (24, 25). We hypothesize that surgical excision of the tonsils may disrupt this homing axis, leading to compensatory but dysregulated recruitment of lymphocytes to the salivary glands, a hallmark of SS pathogenesis.

To address these gaps, we used a large-scale, real-world evidence platform—the TriNetX US Collaborative Network—to investigate whether tonsillectomy or adenoidectomy is associated with an increased long-term risk of developing SS. Using millions of electronic health records across diverse healthcare systems, we conducted a propensity score–matched cohort study designed to minimize confounding and provide robust, population-level estimates. We further explored effect modification by demographic and clinical subgroups and conducted negative outcome control analyses to assess the specificity of observed associations. By integrating immunologic rationale with contemporary epidemiologic methodology, this study seeks to clarify whether surgical removal of lymphoid tissue contributes to autoimmune susceptibility and to inform clinical decision-making regarding the long-term consequences of tonsillectomy and adenoidectomy.

## 2 Materials and methods

### 2.1 Study design and data source

This study used a retrospective cohort design based on data from the TriNetX US Collaborative Network, a federated real-world evidence platform that aggregates de-identified electronic health records from more than seventy healthcare organizations across the United States. The network encompasses diverse healthcare settings, including tertiary academic medical centers, regional hospitals, and ambulatory care systems, thereby providing a broad representation of demographic and clinical characteristics. The platform captures detailed data elements such as diagnostic and procedural codes, medication prescriptions, laboratory measurements, and longitudinal encounter histories. All analyses were conducted within the secure TriNetX environment, which returns only aggregated, non-identifiable data and automatically enforces privacy protections. The study protocol was reviewed and approved by the institutional review board of Chung Shan Medical University Hospital (CS2-21176). Written informed consent from participants was waived because data were obtained from a de-identified database.

### 2.2 Study population and index date

Patients were eligible for inclusion if they had a diagnosis of tonsillar or adenoidal disorders between January 1, 2006, and December 31, 2023, identified using *ICD-10-CM* codes J03 (acute tonsillitis), J35 (chronic diseases of tonsils and adenoids), or J36 (peritonsillar abscess). The first recorded diagnosis of tonsillar or adenoidal disorders (*ICD-10-CM* J03, J35, or J36) was defined as the index event for cohort entry. Individuals were assigned to the surgical cohort if they underwent tonsillectomy or adenoidectomy within 90 days of the index diagnosis, as identified by relevant Current Procedural Terminology (CPT) and *ICD-10-PCS* procedure codes. Patients with the same diagnoses but without any recorded history of tonsillar or adenoidal surgery were assigned to the non-surgical cohort. Individuals in the non-surgical cohort were censored at the time of subsequent tonsillectomy or adenoidectomy if such surgery occurred beyond the initial 90-day exposure window. To ensure adequate observation, patients were required to have at least 12 months of medical records both before and after the index date. Individuals who had undergone tonsillectomy or adenoidectomy prior to the index diagnosis were excluded to maintain clear exposure classification. Additional exclusions were applied to remove patients with a documented history of SS (*ICD-10-CM*: M35.0), other systemic autoimmune diseases, clinically significant immunodeficiency, or incomplete demographic information necessary for covariate adjustment. Patients with inadequate follow-up duration or missing essential laboratory and medication data required for propensity score modeling were also excluded.

### 2.3 Exposure definition

The exposure group comprised patients who underwent tonsillectomy or adenoidectomy within 3 months of the index diagnosis, as identified using standardized CPT and Systematized Nomenclature of Medicine Clinical Terms (SNOMED CT) procedure codes (**Supplementary Table S1**). The 90-day interval was selected to capture procedures performed as part of clinical management for tonsillar or adenoidal disorders rather than unrelated surgical interventions. Patients without any of these procedures served as the comparison group. The temporal alignment of exposure relative to diagnosis allowed for standardized follow-up and reduced misclassification. Individuals with prior tonsillar/adenoidal surgery before the index diagnosis were excluded to ensure clear exposure assignment.

### 2.4 Outcome definition

The primary outcome was incident SS, identified by the first occurrence of *ICD-10-CM* code M35.0 after the index date. Both surgical and non-surgical patients were followed from the index date until the earliest of outcome diagnosis, death, loss to follow-up, or the end of available data (December 31, 2023).

### 2.5 Covariates and propensity score matching

To minimize baseline differences and improve comparability between the surgical and non-surgical cohorts, propensity score matching was performed using TriNetX’s built-in nearest-neighbor algorithm with a caliper of 0.1 pooled standard deviations. Propensity scores were estimated based on a comprehensive set of covariates, including demographic factors such as age, sex, race, and ethnicity; clinical factors such as body mass index (BMI), smoking history, and comorbidity profiles; healthcare utilization metrics; and specific tonsillar or adenoidal disorder subtypes (**Supplementary Table S2**). Adequacy of covariate balance between groups was assessed using standardized mean differences, with values below 0.1 indicating acceptable balance for all matched variables.

### 2.6 Statistical analysis

All analyses were performed using the matched cohort. Descriptive statistics summarized baseline characteristics, with continuous variables presented as means and standard deviations (SDs), and categorical variables as frequencies and percentages. Time-to-event analyses were conducted using Cox proportional hazards modeling to estimate hazard ratios and 95% confidence intervals for the association between tonsillectomy or adenoidectomy and incident SS. Cumulative incidence was estimated using Kaplan–Meier survival analysis, and group differences were compared using the log-rank test. Subgroup analyses examined potential effect modification by age group (<18, 18–64, and ≥65 years), sex, race and ethnicity, and BMI categories (<25 and ≥25 kg/m²). Age categories were defined *a priori* to distinguish pediatric and adolescent immune development (<18 years) from adulthood, consistent with established principles of mucosal immune maturation.

Sensitivity analyses were performed to evaluate the robustness of the study findings and to assess the potential influence of unmeasured confounding. First, a series of negative outcome control analyses were conducted by examining the association between tonsillectomy or adenoidectomy and several clinical conditions unlikely to share causal pathways with SS. These control outcomes included burns, toxic exposures, smoke or fire-related injuries, gout, and malignant neoplasms (**Supplementary Table S3**). Second, the temporal relationship between the exposure and outcome was further tested by applying alternative definitions of the exposure window. Specifically, the interval during which tonsillectomy or adenoidectomy was considered relevant to the index diagnosis was extended from the primary 3-month definition to 6-month and 12-month post-index periods. All tests were two-tailed, and a P value <0.05 was considered statistically significant. Statistical analyses were conducted using the built-in analytics functions of the TriNetX platform (TriNetX, LLC, Cambridge, MA, USA).

## 3 Results

### 3.1 Study population

**Figure 1:**
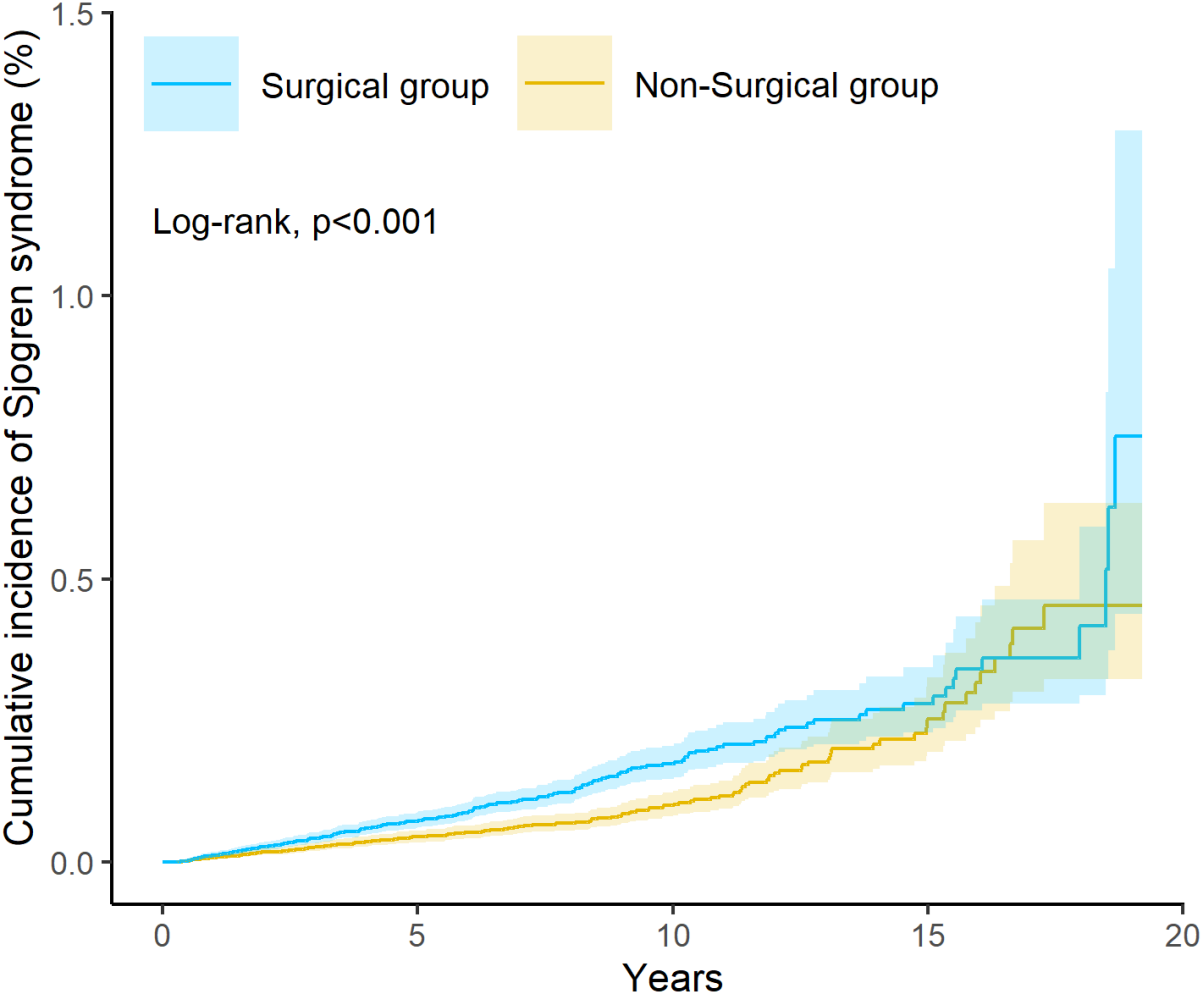
Study flow diagram illustrating patient identification and propensity score matching process. CRP, C reactive protein; ESR, erythrocyte sedimentation rate.

A total of 2,720,314 patients with diagnoses related to tonsillar or adenoidal disorders were identified within the TriNetX US Collaborative Network between January 2006 and December 2023. Among these, 311,832 individuals who underwent tonsillectomy or adenoidectomy were classified as the surgical cohort. After 1:1 propensity score matching, 302,737 patients were successfully included in each group—the surgical and non-surgical cohorts—resulting in a total of 605,474 matched participants (**Figure 1**).

Baseline demographic, socioeconomic, and clinical characteristics were well balanced between the two matched cohorts, with standardized mean differences less than 0.1 across all covariates (**Table 1**). The distributions of age, sex, race, comorbidities, medication use, and laboratory indices were comparable.

**Table 1:** Baseline demographic, clinical, and laboratory characteristics of surgical and non-surgical group before and after matching.

Demographic, clinical, and laboratory characteristics	Before PSM		SMD	After PSM		SMD
	Surgical group N = 311718	Non-Surgical group N = 2227037		Surgical group N = 302737	Non-Surgical group N = 302737	
Age, Mean ± SD	9.36 ± 10.53	19.50 ± 16.96	0.718	9.51 ± 10.63	9.62 ± 10.68	0.010
Sex						
Female	149220 (47.87)	1218001 (54.69)	0.137	145741 (48.14)	145522 (48.07)	0.001
Male	158005 (50.69)	964684 (43.32)	0.148	152514 (50.38)	152767 (50.46)	0.002
Unknown Gender	4493 (1.44)	44352 (1.99)	0.042	4482 (1.48)	4448 (1.47)	0.001
Race						
White	196050 (62.89)	1366698 (61.37)	0.031	190672 (62.98)	190937 (63.07)	0.002
Black or African American	45201 (14.50)	336865 (15.13)	0.018	43200 (14.27)	41603 (13.74)	0.015
Asian characteristic(s)	6790 (2.18)	66533 (2.99)	0.051	6686 (2.21)	6853 (2.26)	0.004
Native Hawaiian or Other Pacific Islander	1409 (0.45)	12720 (0.57)	0.017	1385 (0.46)	1427 (0.47)	0.002
American Indian or Alaska Native	1949 (0.63)	10516 (0.47)	0.021	1890 (0.62)	1378 (0.46)	0.023
Other Race	19754 (6.34)	145856 (6.55)	0.009	19116 (6.31)	21806 (7.20)	0.035
Unknown Race	40565 (13.01)	287849 (12.93)	0.003	39788 (13.14)	38733 (12.79)	0.010
**BMI, Mean ± SD**	21.61 ± 7.88	25.91 ± 8.69	0.518	21.75 ± 7.96	21.83 ± 8.18	0.009
<25 kg/m^2^	57077 (18.31)	342498 (15.38)	0.078	53937 (17.82)	51744 (17.09)	0.019
≥25 kg/m^2^	21194 (6.80)	317889 (14.27)	0.245	20691 (6.84)	19749 (6.52)	0.012
Social economic status						
Persons with potential health hazards related to socioeconomic and psychosocial circumstances	3109 (1.00)	19146 (0.86)	0.014	2880 (0.95)	2557 (0.85)	0.011
Housing/economic circumstances problem	267 (0.09)	3368 (0.15)	0.019	254 (0.08)	265 (0.09)	0.001
Problems related to education and literacy	293 (0.09)	2045 (0.09)	0.001	274 (0.09)	314 (0.10)	0.004
Medical utilization						
Ambulatory	168778 (54.14)	1274249 (57.22)	0.062	161962 (53.50)	161225 (53.26)	0.005
Emergency	39950 (12.82)	340740 (15.30)	0.072	38693 (12.78)	37050 (12.24)	0.016
Inpatient Encounter	12161 (3.90)	113388 (5.09)	0.057	11466 (3.79)	11336 (3.75)	0.002
Comorbidities						
Nicotine dependence	1786 (0.57)	46062 (2.07)	0.131	1786 (0.59)	1838 (0.61)	0.002
Alcohol related disorders	293 (0.09)	8679 (0.39)	0.060	292 (0.10)	292 (0.10)	0.000
Overweight and obesity	10377 (3.33)	97627 (4.38)	0.055	9755 (3.22)	9143 (3.02)	0.012
Hypertension	4192 (1.35)	99166 (4.45)	0.186	4045 (1.34)	4675 (1.54)	0.017
Hyperlipidemia	1867 (0.60)	52964 (2.38)	0.147	1838 (0.61)	2220 (0.73)	0.015
Chronic kidney disease	669 (0.22)	10432 (0.47)	0.044	624 (0.21)	696 (0.23)	0.005
Asthma	20167 (6.47)	130782 (5.87)	0.025	18923 (6.25)	17584 (5.81)	0.019
Allergic rhinitis	15083 (4.84)	90693 (4.07)	0.037	14236 (4.70)	13065 (4.32)	0.019
Obstructive sleep apnea	31424 (10.08)	49730 (2.23)	0.331	22443 (7.41)	23115 (7.64)	0.008
Other anxiety disorders	6360 (2.04)	105421 (4.73)	0.149	6285 (2.08)	5844 (1.93)	0.010
Juvenile arthritis	174 (0.06)	994 (0.05)	0.005	168 (0.06)	161 (0.05)	0.001
Rheumatoid arthritis with rheumatoid factor	39 (0.01)	1243 (0.06)	0.023	39 (0.01)	55 (0.02)	0.004
Other rheumatoid arthritis	189 (0.06)	4240 (0.19)	0.037	188 (0.06)	208 (0.07)	0.003
Systemic lupus erythematosus	104 (0.03)	1702 (0.08)	0.018	102 (0.03)	99 (0.03)	0.001
Medications						
Corticosteroids for systemic use	48278 (15.49)	316558 (14.21)	0.036	45729 (15.11)	44620 (14.74)	0.010
Anti-inflammatory and antirheumatic products, non-steroids	38021 (12.20)	293728 (13.19)	0.030	36292 (11.99)	34707 (11.46)	0.016
Antibacterials for systemic use	85222 (27.34)	605234 (27.18)	0.004	82205 (27.15)	79561 (26.28)	0.020
Antihistamines for systemic use	45061 (14.46)	251363 (11.29)	0.095	42132 (13.92)	40411 (13.35)	0.017
Laboratory						
C-reactive protein (mg/L)	23.96 ± 39.55	22.89 ± 43.31	0.026	23.74 ± 39.39	27.07 ± 43.82	0.080
Erythrocyte sedimentation rate (mm/h)	17.79 ± 18.29	18.87 ± 20.01	0.057	17.84 ± 18.34	19.02 ± 20.58	0.061
Leukocytes [#/volume] in Blood (10*3/uL)	10.19 ± 73.62	10.80 ± 102.30	0.007	10.25 ± 75.42	10.65 ± 85.65	0.005
HbA1c (%)	5.90 ± 1.69	6.11 ± 1.66	0.122	5.91 ± 1.70	5.99 ± 1.66	0.045

PSM, Propensity socre matching; SMD, Standardized mean difference.

### 3.2 Incidence of Sjögren’s syndrome

**Figure 2:**
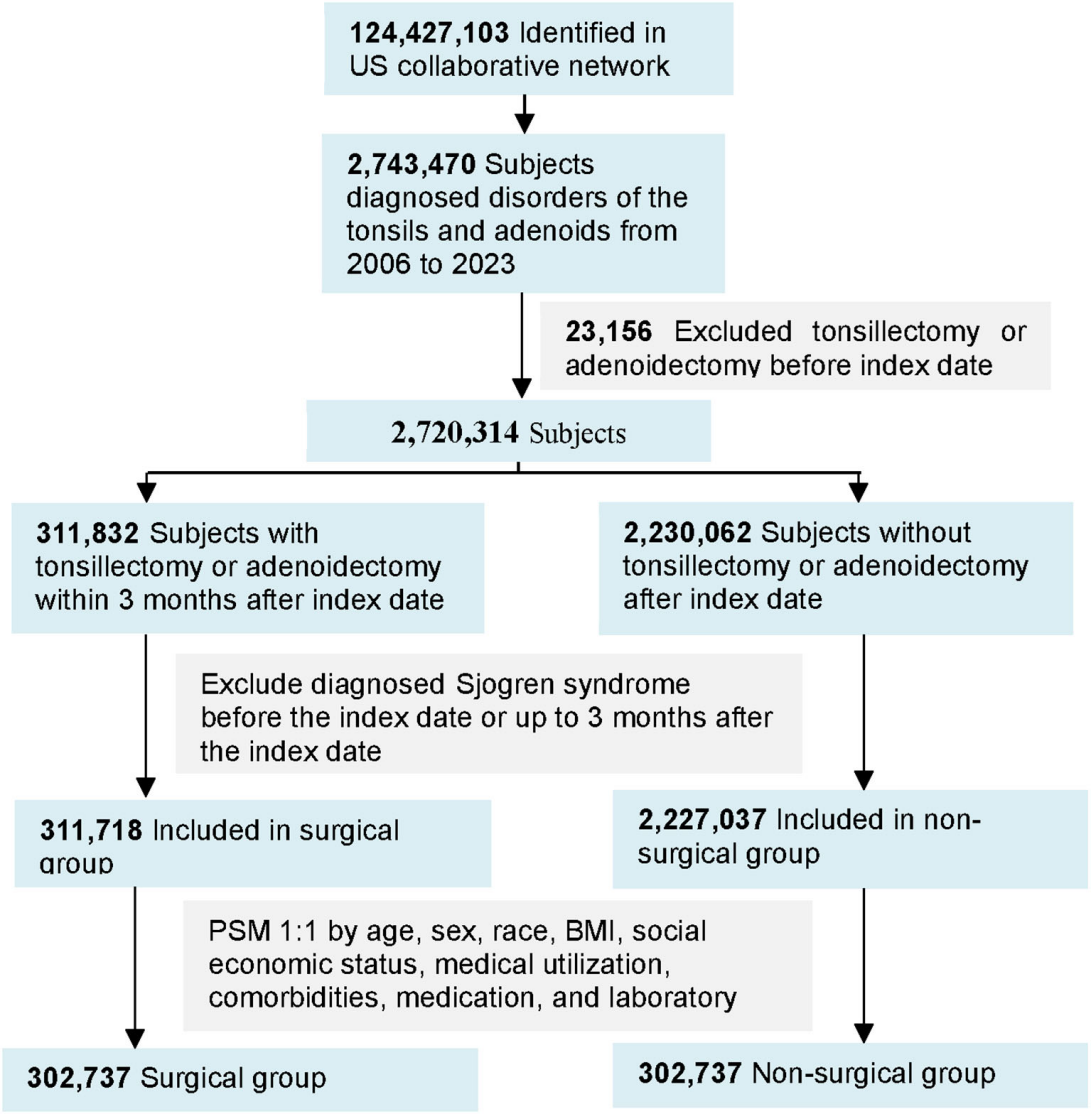
Kaplan–Meier curves showing cumulative incidence of Sjögren’s syndrome in surgical versus non-surgical cohorts.

During the observation period, 213 patients in the surgical cohort and 153 in the non-surgical cohort were newly diagnosed with SS. The corresponding cumulative incidence rates were 1.32% and 0.45%, respectively, corresponding to an absolute risk increase of approximately 0.87% over long-term follow-up. Kaplan–Meier survival analysis demonstrated a significantly higher cumulative incidence of SS among patients who underwent tonsillectomy or adenoidectomy compared with those who did not (P < 0.001 by log-rank test; **Figure 2**). The widening confidence intervals at later time points in **Figure 2** reflect decreasing numbers at risk, a common feature of long-term survival analyses.

The proportional hazards assumption for the Cox model was assessed, and no evidence of violation was observed (*P* for proportional hazards assumption = 0.101; **Table 2**). In the Cox proportional hazards model, tonsillectomy or adenoidectomy was associated with a 52% increased risk of developing SS (HR = 1.52; 95% CI: 1.23–1.87; **Table 2**). These results suggest that removal of tonsillar or adenoidal tissue may confer an elevated long-term susceptibility to autoimmune dysfunction.

**Table 2:** Risk of Sjögren’s syndrome comparing surgical group to non-surgical group.

Outcome measure	Surgical group (N = 302,737)	Non-Surgical group (N = 302,737)	*P* for proportional hazard assumption
No. of event	213	153	
Cumulative incidence (%)	1.32	0.45	
HR (95% C.I.)	1.52 (1.23–1.87)	Reference	0.101

### 3.3 Subgroup analyses

**Figure 3:**
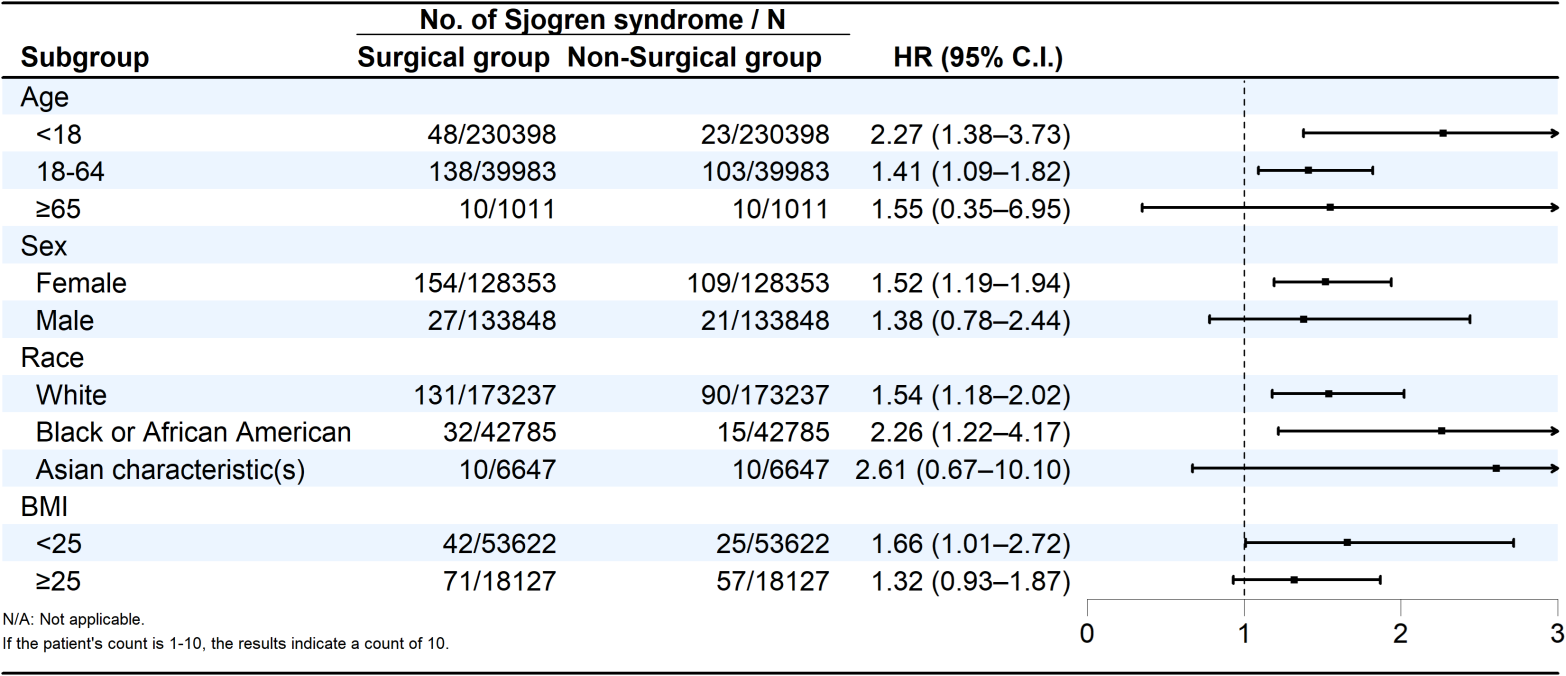
Forest plot of subgroup-specific hazard ratios with 95% confidence intervals.

Subgroup analyses stratified by age, sex, race, and BMI showed a markedly elevated risk among patients younger than 18 years, with an HR of 2.27 (95% CI: 1.38–3.73), indicating that tonsillectomy during childhood or adolescence may disrupt mucosal immune regulation and predispose to autoimmunity later in life (**Figure 3**). Similarly, African American patients exhibited a significantly increased risk (HR = 2.26; 95% CI: 1.22–4.17).

Among Asian patients, the hazard ratio suggested a possible upward trend (HR = 2.61), though the confidence interval (95% CI: 0.67–10.10) did not reach statistical significance, potentially reflecting smaller subgroup size. Across BMI categories, individuals with BMI < 25 kg/m² demonstrated a significantly higher risk (HR = 1.66; 95% CI: 1.01–2.72).

When stratified by sex, female patients showed a significant association (HR = 1.52; 95% CI: 1.19–1.94), whereas male patients did not (HR = 1.38; 95% CI: 0.78–2.44). No significant interaction was observed between sex and surgical exposure.

### 3.4 Sensitivity analyses

To evaluate the robustness of the primary findings, sensitivity analyses were conducted using negative outcome controls involving conditions unrelated to tonsillectomy or autoimmune disease. None of these outcomes demonstrated statistically significant associations with tonsillectomy or adenoidectomy (all HRs ≈ 1.0; **Table 3**), indicating the absence of systematic bias or residual confounding.

**Table 3:** Sensitivity analyses using negative outcome controls.

Negative outcome control	Surgical group		Non-surgical group		HR (95% C.I.)
	N	No. of event	N	No. of event	
Negative outcome control					
Burn	300,976	1,586	300,976	1,750	0.98 (0.92–1.05)
Toxic effects of non-medicinal substances	300,205	2,341	300,205	2,669	0.95 (0.90–1.00)
Exposure to smoke, fire and flames	266,716	106	266,716	121	0.95 (0.73–1.23)
Gout	302,557	298	302,557	312	1.04 (0.89–1.22)
Cancer	263,639	1,057	263,639	1,051	1.09 (0.999–1.19)

Additional sensitivity analyses assessing alternative definitions of the exposure window (extending to 6- and 12-month post-index periods) yielded comparable effect estimates, further confirming the stability of the observed association. Collectively, these findings reinforce the internal validity and temporal consistency of the results.

## 4 Discussion

In this large, multicenter, real-world cohort study of more than 2.7 million individuals across the United States, we observed that tonsillectomy or adenoidectomy was associated with a significantly increased long-term risk of developing SS. This association persisted after rigorous propensity score matching, was consistent across multiple analytic approaches, and remained robust in sensitivity analyses and negative outcome control assessments. The absence of a similar association with unrelated conditions (e.g., burns, trauma) supports that the observed link is unlikely to be explained by differential healthcare utilization or baseline frailty. Notably, the elevated risk was most pronounced among younger patients and African American individuals, suggesting possible age- and ancestry-related differences in immunologic vulnerability. Collectively, these findings offer new insight into the potential autoimmune consequences of surgically disrupting mucosal lymphoid tissues. Despite the statistically significant association, the absolute risk of SS remains low. Therefore, we reported the absolute risk difference (0.87%) between surgical and non-surgical cohorts to contextualize these findings and avoid clinical overinterpretation.

The observed association is biologically plausible considering the well-established immunologic functions of tonsils and adenoids (26). These structures are central components of the mucosa-associated lymphoid tissue system and serve as primary inductive sites where local antigens are sampled and presented to the immune system (27). They host specialized epithelial and lymphoid microenvironments conducive to antigen uptake, germinal center formation, affinity maturation, B-cell differentiation, and IgA class-switch recombination. During infancy, childhood, and adolescence, these tissues also play a pivotal role in shaping immune tolerance by promoting balanced interactions between T-follicular helper cells, regulatory T cells, and mucosal stromal elements (26). Removal of these tissues may therefore perturb the finely tuned processes that regulate oral and upper aerodigestive mucosal immunity, potentially shifting developmental immune trajectories toward heightened autoreactivity. Furthermore, disruption of this microenvironment through surgical excision may disturb B-cell maturation, germinal center formation (28), and regulatory T-cell homeostasis, predisposing individuals to maladaptive immune responses (29). Previous histopathological studies have demonstrated diminished germinal-center activity and reduced CD10^+^ B-cell populations following tonsillectomy in children (29), while others have noted partial compensatory responses by residual oropharyngeal lymphoid tissue (30, 31). Our study extends these mechanistic insights by demonstrating that such immunologic disruption may translate into clinically measurable, long-term autoimmune risk (12).

A common concern in observational surgical epidemiology is reverse causality, namely that the inflammatory or immune predisposition leading to tonsillectomy may itself represent an early manifestation of autoimmune susceptibility. Several features of our data argue against this explanation as the sole driver of the observed association. Most notably, the Kaplan–Meier curves demonstrate a gradual and progressive divergence in SS incidence over long-term follow-up, rather than an early postoperative spike. This temporal pattern is inconsistent with detection bias, perioperative immune activation, or immediate reverse causation. Furthermore, the prolonged latency between surgical exposure and outcome is consistent with the natural history of autoimmune disease development and supports a model in which early-life disruption of mucosal immune education precedes, rather than reflects, later autoimmune manifestation. While causality cannot be definitively established in an observational design, these temporal dynamics, together with extensive propensity score matching and negative outcome control analyses, strengthen the plausibility of a directional association from lymphoid tissue removal to subsequent autoimmune risk.

The age-stratified findings warrant emphasis. The <18-year threshold was selected to reflect a critical developmental window in which tonsillar and adenoidal tissues play a central role in immune education, tolerance induction, and imprinting of mucosal B-cell responses. During childhood and adolescence, Waldeyer’s ring functions as a key inductive site for IgA class switching, regulatory T-cell expansion, and establishment of long-term mucosal immune homeostasis. Surgical removal of these tissues during this period may therefore have disproportionate and enduring effects on immune regulation compared with removal in adulthood, when mucosal immune networks are more mature and potentially compensatory. The markedly higher hazard ratio observed among individuals undergoing surgery before age 18 is consistent with this developmental immunology framework and supports the hypothesis that early-life disruption of mucosal immune education increases long-term susceptibility to epithelial-targeted autoimmunity such as SS.

The pronounced susceptibility among younger patients also lends support to the concept that tonsillar tissue contributes to immune education and peripheral tolerance establishment during critical developmental windows (5, 31). Removal of these lymphoid structures may hinder the normal maturation of immune regulation, particularly toward epithelial antigens expressed in salivary and lacrimal glands—the hallmark targets of SS (15). The over two-fold risk observed in African American patients (HR 2.26) represents a novel and clinically significant finding. This racial disparity likely reflects a complex interplay between ancestry-specific genetic polymorphisms and environmental triggers. African American populations are known to carry distinct risk alleles in the *HLA-DRB1* and *IRF5* loci, which are strongly associated with SS susceptibility and B-cell hyperactivation. We hypothesize that the surgical removal of tonsillar tissue—a key environmental “hit” to the mucosal immune system—may act synergistically with these genetic backgrounds, lowering the threshold for tolerance breakdown (32). This underscores the need for precision surveillance in this demographic, as they may be uniquely vulnerable to the long-term immunologic sequelae of lymphoid tissue excision.

Evidence supporting this hypothesis has been accumulating. Previous large-scale epidemiologic studies have demonstrated associations between tonsillectomy and several autoimmune diseases, including inflammatory bowel disease, autoimmune thyroiditis, multiple sclerosis, and rheumatoid arthritis (8, 11). While these findings do not establish causation, they suggest that lymphoid excision may have long-term systemic immunologic consequences that extend beyond the oropharyngeal region. SS, characterized by lymphocytic infiltration of exocrine glands and widespread epithelial–immune dysregulation, fits within this framework of diseases potentially influenced by early-life immune disruption. Aberrant B-cell activation, germinal center-like activity within salivary gland tissues, and dysregulated type I interferon signaling are core features of SS (17, 18); these pathways may be sensitized or inadequately regulated when mucosal lymphoid organs are surgically removed at critical developmental periods.

Although the present study is observational and cannot establish mechanistic causality, our findings align with the “mucosal bridge” hypothesis, suggesting that the tonsils and salivary glands are functionally linked. The palatine tonsils act as a primary inductive site where naive B cells are exposed to oral antigens and differentiate into IgA-producing plasmablasts. Under normal homeostasis, these cells traffic to effector sites, including the salivary and lacrimal glands, to provide immune exclusion. We propose two primary mechanisms for the observed association. First, loss of homing regulation: Without tonsillar guidance, B-cells may undergo aberrant activation in secondary lymphoid organs and mis-home to the salivary epithelium. The removal of tonsillar stromal cells—which regulate lymphocyte trafficking—could lead to compensatory but dysregulated recruitment of lymphocytes to the salivary glands, initiating the focal lymphocytic sialadenitis characteristic of SS. Second, microbiota dysbiosis: The tonsils host a specific crypt microbiota that regulates T-regulatory cell expansion (33). Their removal has been shown to alter the oropharyngeal microbiome and may disrupt the mucosal barrier integrity (34). Given that dysbiosis is a known driver of SS, the surgical alteration of the oral commensal landscape may lower the threshold for autoimmune activation by influencing systemic T- and B-cell regulation.

These results also carry important clinical implications. Tonsillectomy and adenoidectomy remain widely performed procedures, especially in children. Although the absolute risk increase for SS is modest, the findings underscore the importance of carefully weighing the long-term immunologic consequences of surgical lymphoid tissue removal, particularly in individuals with personal or family histories of autoimmunity. Clinicians may consider enhanced surveillance for early signs of sicca symptoms, parotid swelling, fatigue, or autoimmune serologic markers in patients with prior tonsillar or adenoidal surgery. The results do not argue against tonsillectomy when it is clearly indicated—for example, in cases of clinically significant obstructive sleep apnea or severe recurrent infection—but they do suggest that its long-term immune impact should not be overlooked.

Interestingly, not all studies have associated tonsillectomy with adverse immune outcomes. In some autoimmune conditions such as IgA nephropathy, palmoplantar pustulosis, and psoriasis, tonsillectomy has been shown to alleviate disease activity by reducing chronic antigenic stimulation and downregulating aberrant immune signaling (12, 35). This apparent paradox highlights the context-dependent duality of tonsillar immunity: whereas persistent antigenic drive can perpetuate inflammation in established autoimmunity, removal of organized lymphoid tissue in otherwise immunocompetent individuals may impair immune tolerance and foster new-onset autoimmunity. Therefore, the immunologic consequence of tonsillectomy is not uniform but influenced by surgical timing, baseline immune status, and the capacity for mucosal immune reconstitution following surgery (36, 37).

From a clinical standpoint, these findings carry meaningful implications. Tonsillectomy remains a safe and effective procedure for recurrent tonsillitis, peritonsillar abscess, and obstructive sleep-disordered breathing. Nonetheless, our results suggest that the procedure may have long-term systemic immune ramifications for a subset of patients. Awareness of this potential association should prompt clinicians to consider individualized preoperative risk assessment, particularly for young or immunologically susceptible patients. Postoperative surveillance might include education regarding dryness-related symptoms, fatigue, or other early indicators of autoimmune dysfunction. Future studies integrating longitudinal immunophenotyping, cytokine profiling, and mucosal microbiome analyses will be instrumental in elucidating how surgical disruption of the tonsillar immune niche contributes to exocrine-gland autoimmunity.

### 4.1 Limitations

Several limitations warrant acknowledgement. First, as with any observational study, residual confounding and reverse causality cannot be fully excluded. Although the long latency and absence of an early postoperative risk spike argue against immediate detection bias or perioperative effects, unmeasured factors may influence both the likelihood of tonsillectomy/adenoidectomy and subsequent autoimmune risk. Second, outcome ascertainment relies on *ICD* coding, and diagnostic practices vary across institutions; thus, SS diagnoses may not perfectly align with standardized classification criteria, introducing potential misclassification. Third, the TriNetX dataset lacks granular clinical phenotyping and treatment response, and it does not capture key mechanistic parameters (e.g., mucosal microbiome shifts or immunophenotypic changes) needed to directly test causal pathways. Fourth, some subgroup analyses—particularly by sex—may have been underpowered given the low absolute incidence of SS in men; therefore, the absence of a statistically significant sex–exposure interaction should not be interpreted as evidence of biological equivalence. Fifth, procedure coding may introduce exposure heterogeneity; for example, inclusion of uvulopalatopharyngoplasty may variably entail tonsillar excision and could contribute to exposure misclassification (38, 39). Finally, reliance on healthcare utilization data may lead to under-ascertainment of SS, especially in mild or early-stage cases with delayed diagnosis. Despite these constraints, the findings provoke compelling questions. Future longitudinal cohort studies integrating immunoprofiling, genomic risk scores, and mucosal microbiome analyses are needed to clarify whether lymphoid removal triggers immune reprogramming or merely unmasks preexisting susceptibility. Mechanistic studies utilizing tonsillar tissue, salivary gland organoids, and *in vivo* models could elucidate how early lymphoid removal impacts B-cell selection, T-cell help, and epithelial immune crosstalk. Parallel population-based studies should investigate whether postoperative interventions, such as microbiome modulation, could mitigate long-term autoimmune risk. Finally, the observed racial differences underscore an urgent need to address disparities in autoimmune research, ensuring minority populations are adequately represented in translational studies.

## 5 Conclusion

In summary, this study demonstrates that tonsillectomy and adenoidectomy are associated with a modest but significant increase in the long-term risk of SS, particularly among younger and African American individuals. These findings highlight the enduring immunologic importance of mucosal lymphoid tissues and raise thoughtful questions about the potential systemic consequences of their removal. While further mechanistic and prospective research is needed, the present results contribute meaningfully to our understanding of autoimmune disease pathogenesis and reinforce the importance of integrating immunologic considerations into surgical decision-making.

## Data Availability

The original contributions presented in the study are included in the article/**Supplementary Material**. Further inquiries can be directed to the corresponding author/s.
