# Supplementary material for: Surgical removal of Waldeyer’s ring and long-term risk of Sjögren’s syndrome: a population-based cohort study investigating the mucosal immune link
Source: Front Immunol. 2026 Feb 5;17:1760386. doi: 10.3389/fimmu.2026.1760386 (PMC12916558; doi:10.3389/fimmu.2026.1760386)
Supplement: Supplementary file 1 [file Table1.docx]

**Supplementary Materials**

**Table S1**: The codes for tonsillectomy and adenoidectomy.

**Table S2**: The diagnostic codes (*ICD-10-CM* codes) for the covariates.

**Table S3**: The diagnostic codes (*ICD-10-CM* codes) for the negative control outcomes included burns, toxic exposures, smoke or fire-related injuries, gout, and malignant neoplasms.

**Table S1**: The codes for tonsillectomy and adenoidectomy.

| **Procedure** | **Code** |
| --- | --- |
| Tonsillectomy and adenoidectomy | CPT:1007178 |
| Tonsillectomy | SNOMED:173422009 |
| Tonsillectomy and adenoidectomy | CPT:1007178 |
| Tonsillectomy and adenoidectomy; younger than age 12 | CPT:42820 |
| Tonsillectomy, primary or secondary | CPT:1007181 |
| Tonsillectomy, primary or secondary; age 12 or over | CPT:42826 |
| Tonsillectomy and adenoidectomy; age 12 or over | CPT:42821 |
| Tonsillectomy, primary or secondary; younger than age 12 | CPT:42825 |
| Tonsillectomy and adenoidectomy | SNOMED:28913000 |
| Adenoidectomy, primary | CPT:1007184 |
| Adenoidectomy, primary; younger than age 12 | CPT:42830 |
| Adenoidectomy, primary; age 12 or over | CPT:42831 |
| Adenoidectomy, secondary | CPT:1007187 |
| Adenoidectomy, secondary; younger than age 12 | CPT:42835 |
| Adenoidectomy, secondary; age 12 or over | CPT:42836 |
| Tonsillectomy and adenoidectomy | CPT:1007178 |
| Tonsillectomy and adenoidectomy; younger than age 12 | CPT:42820 |
| Tonsillectomy and adenoidectomy; age 12 or over | CPT:42821 |
| Tonsillectomy and adenoidectomy | SNOMED:28913000 |

CPT: Current Procedural Terminology.

SNOMED: Systematic Nomenclature of Medicine.

**Table S2**: The diagnostic codes (*ICD-10-CM* codes) for the covariates.

| **Covariates** | ***ICD-10-CM* codes** |
| --- | --- |
| **Social economic status** |  |
| Persons with potential health hazards related to socioeconomic and psychosocial circumstances | Z55-Z65 |
| Housing/economic circumstances problem | Z59 |
| Problems related to education and literacy | Z55 |
| **Comorbidities** |  |
| Nicotine dependence | F17 |
| Alcohol related disorders | F10 |
| Overweight and obesity | E66 |
| Hypertension | I10 |
| Hyperlipidemia | E78.5 |
| Chronic kidney disease | N18 |
| Asthma | J45 |
| Allergic rhinitis | J30.9 |
| Obstructive sleep apnea | G47.33 |
| Other anxiety disorders | F41 |
| Juvenile arthritis | M08 |
| Rheumatoid arthritis with rheumatoid factor | M05 |
| Other rheumatoid arthritis | M06 |
| Systemic lupus erythematosus | M32 |
| **Medications** |  |
| Corticosteroids for systemic use | ATC: H02 |
| Anti-inflammatory and antirheumatic products, non-steroids | ATC: M01A |
| Antibacterials for systemic use | ATC: J01 |
| Antihistamines for systemic use | ATC: R06 |
| **Laboratory** |  |
| C-reactive protein (mg/L) | TNX: 9063 |
| Erythrocyte sedimentation rate (mm/h) | TNX: 9066 |
| Leukocytes [#/volume] in Blood (10*3/uL) | TNX: 9015 |
| HbA1c (%) | TNX: 9037 |

ICD-10-CM: International Classification of Diseases, Tenth Revision, Clinical Modification.

ATC: Anatomical Therapeutic Chemical.

TNX: TrinetX curated.

**Table S3**: The diagnostic codes (*ICD-10-CM* codes) for the negative control outcomes included burns, toxic exposures, smoke or fire-related injuries, gout, and malignant neoplasms.

| **Burn (T20-T25):**  T20 Burn and corrosion of head, face, and neck  T21 Burn and corrosion of trunk  T22 Burn and corrosion of shoulder and upper limb, except wrist and hand  T23 Burn and corrosion of wrist and hand  T24 Burn and corrosion of lower limb, except ankle and foot  T25 Burn and corrosion of ankle and foot |
| --- |
| **Toxic effects of non-medicinal substances (T51-T65):**  T51 Toxic effect of alcohol  T52 Toxic effect of organic solvents  T53 Toxic effect of halogen derivatives of aliphatic and aromatic hydrocarbons  T54 Toxic effect of corrosive substances  T55 Toxic effect of soaps and detergents  T56 Toxic effect of metals  T57 Toxic effect of other inorganic substances  T58 Toxic effect of carbon monoxide  T59 Toxic effect of other gases, fumes and vapors  T60 Toxic effect of pesticides  T61 Toxic effect of noxious substances eaten as seafood  T62 Toxic effect of other noxious substances eaten as food  T63 Toxic effect of contact with venomous animals and plants  T64 Toxic effect of aflatoxin and other mycotoxin food contaminants  T65 Toxic effect of other and unspecified substances |
| **Exposure to smoke, fire and flames (X00-X08):**  X00 Exposure to uncontrolled fire in building or structure  X01 Exposure to uncontrolled fire, not in building or structure  X02 Exposure to controlled fire in building or structure  X03 Exposure to controlled fire, not in building or structure  X04 Exposure to ignition of highly flammable material  X05 Exposure to ignition or melting of nightwear  X06 Exposure to ignition or melting of other clothing and apparel  X08 Exposure to other specified smoke, fire and flames |
| **Gout**  M10 Gout |
| **Cancer**  C00-C14 Malignant neoplasms of lip, oral cavity and pharynx  C15-C26 Malignant neoplasms of digestive organs  C30-C39 Malignant neoplasms of respiratory and intrathoracic organs  C40-C41 Malignant neoplasms of bone and articular cartilage  C43-C44 Melanoma and other malignant neoplasms of skin  C45-C49 Malignant neoplasms of mesothelial and soft tissue  C50-C50 Malignant neoplasms of breast  C51-C58 Malignant neoplasms of female genital organs  C60-C63 Malignant neoplasms of male genital organs  C64-C68 Malignant neoplasms of urinary tract  C69-C72 Malignant neoplasms of eye, brain and other parts of central nervous system  C73-C75 Malignant neoplasms of thyroid and other endocrine glands  C76-C80 Malignant neoplasms of ill-defined, other secondary and unspecified sites  C7A-C7A Malignant neuroendocrine tumors  C7B-C7B Secondary neuroendocrine tumors  C81-C96 Malignant neoplasms of lymphoid, hematopoietic and related tissue |
